# Knowledge and Attitudes of Medical Students in Palliative Medicine

**DOI:** 10.7759/cureus.95690

**Published:** 2025-10-29

**Authors:** Natasha Seebaran, Victoria Millington, Jason Liu, Estee Hong, Feroza Daroowalla

**Affiliations:** 1 Department of Medical Education, University of Central Florida College of Medicine, Orlando, USA; 2 Department of Internal Medicine, Cleveland Clinic, Weston, USA

**Keywords:** medical student attitudes, medical student knowledge, palliative care, palliative medicine, undergraduate medical education

## Abstract

Background: Primary palliative care (PPC) is a specialized field that focuses mainly on the quality of life through symptom management, goal setting/advanced care planning, and psychosocial support in patients with serious illnesses. However, it is underutilized due to misconceptions about its scope and how to provide it. There remains great variability in the preparation of residents and providers in palliative medicine.

Objective: This study assessed medical students' level of knowledge and attitudes about PPC concepts.

Methods: A survey was adapted from previously validated questionnaires for physicians and nurses to assess knowledge in different domains of palliative medicine and evaluate student exposure, experiences, and attitudes surrounding palliative medicine. This study was conducted in the United States. The survey was administered to the University of Central Florida College of Medicine students in all years of training during the 2023-2024 academic year.

Results: A total of 125 responses from 480 distributed surveys were collected (26% response rate, N = 125). Of these respondents, 39 respondents did not provide their year of training and, thus, received limited analysis, resulting in n = 86 (69%). All students showed the poorest performance in the domains Respiratory and Gastrointestinal Symptoms, and the best performance in Healthcare Disparities and Communication and Cultural Influence. Most respondents agree they would benefit from more PPC education and feel that doctors have a responsibility to help patients prepare for end-of-life care. Furthermore, most students feel unprepared to discuss end-of-life decisions.

Conclusion: Based on our results and review of the current literature, medical students need more education on the treatment of respiratory and gastrointestinal conditions in seriously ill patients and exposure to the practice of PPC during medical school. This will increase the likelihood of offering these resources to patients and feeling comfortable with these concepts as residents and attending physicians. Additionally, this study will contribute to the developing body of knowledge regarding medical student education in palliative medicine, as many studies have not been done on this topic.

## Introduction

Palliative medicine focuses on symptom management, communication, and care coordination for patients with serious illnesses and is increasingly recognized as a core competency for clinicians [1]. With an aging population and increasing patient complexity, proficiency in primary palliative care (PPC) is crucial. PPC includes the basics of symptom management, goals-of-care discussions, and advance care planning [2-4].

Despite this need, studies highlight the underutilization of palliative care specialists in both ICU and community settings because physicians require additional support and training [5,6]. Residents frequently encounter seriously ill patients and must develop PPC competencies early in training. However, notable gaps in PPC education persist across residency programs, with inconsistencies in curricula, content delivery, and evaluation methods [7,8]. In addition to being able to care for individual patients in need of palliative care services, on a wider scale, improvement in palliative care education can serve to improve access to palliative care services through early involvement from a primary care physician before referral to a specialist [9].

Efforts to address these gaps include initiatives such as Aquifer's© Palliative Care Project: Standardizing Primary Palliative Care Education for Medical & Health Professions and Students, launched in January 2022, which established core learning objectives based on national guidelines. This program is a national effort to standardize PPC education for students of medical and health professions, emphasizing key areas like communication, interprofessional teamwork, cultural awareness, and equity in serious illness care. Despite these advancements, barriers remain, including limited faculty expertise, time constraints, and inconsistent integration into undergraduate and residency training [9-11].

The value of these knowledge and skill areas has been well recognized by graduate medical residency programs. A systematic review found considerable variability in PPC education across US residency programs. Key competencies emphasized by US residency programs include communication, symptom management, and end-of-life care. However, formal curricula remain inconsistent, and heterogeneity exists in content and delivery methods [7]. While program leadership generally supports PPC curriculum expansion, barriers such as limited time persist. A scoping review found an increase in PPC educational interventions, particularly in emergency medicine, general surgery, internal medicine, and pediatric residencies. However, the content, delivery methods, and evaluation of these interventions remain varied, with a need for more comprehensive evaluations of behavioral outcomes and clinical impact [8]. In hematology-oncology fellowships, PPC education was often varied and often inadequate, with a need for more effective educational models [10]. Various specialties, including neurology, emergency medicine, and pediatrics, have identified deficiencies in PPC training [12,13].

While graduate program directors acknowledge its importance, undergraduate medical education (UME) continues to lack sufficient exposure, with students receiving minimal training in end-of-life care. Consequently, many students feel unprepared for fundamental PPC tasks such as managing terminal pain, discussing death and bereavement, or guiding patients in goals-of-care discussions [14,15].

This study examines medical students' perspectives, knowledge, skills, and attitudes toward PPC in an allopathic medical school in the United States. Students' understanding of palliative care philosophy, symptom management (pain, respiratory, neuropsychiatric, and gastrointestinal symptoms), communication skills, and cultural considerations were assessed. Students' comfort with delivering PPC and their perceived need for further education in this area were also assessed.

The goal of this study is to highlight both the strengths and deficiencies in current palliative care education in UME. As the focus on these issues increases in graduate medical education (GME), in order to equip physicians with the foundational knowledge, skills, and attitudes necessary to support patients with serious illnesses at all stages of care, it is important to determine how to set new medical graduates up for success during their UME years.

This study was presented as a poster at the Florida Medical Association Annual Meeting on August 3, 2024.

## Materials and methods

This study utilized an online survey hosted on Qualtrics (Qualtrics International Inc., Seattle, WA, US), consisting of 49 questions, designed to be completed anonymously and voluntarily by participants. No incentives were offered for participation. Prior to data collection, approval was obtained from the Institutional Review Board of the University of Central Florida to ensure ethical standards were met, but it was determined to be exempt.

The survey was administered to students enrolled in all four years at the University of Central Florida College of Medicine located in Orlando, Florida, United States. Data collection was conducted over a 14-month period, from October 2023 through December 2024. This period allowed for a broad participation window, ensuring responses from a diverse cross-section of students with varying levels of exposure to palliative care in both academic and clinical settings.

On analysis, the subjects were divided into two categories: preclinical and clinical. The preclinical category included the M1 and M2 students who were in their first and second years of medical school, while the clinical category included the M3 and M4 students who were in their third and fourth years of medical school.

The survey questions were derived from established and validated questionnaires to assess specific domains relevant to palliative medicine. These knowledge-based domains included the Philosophy of Palliative Care, Management of Pain, Respiratory, Neuropsychiatric Disorders, Gastrointestinal Symptoms, and Communication and Cultural Influence. Additionally, the survey aimed to evaluate medical students' exposure to palliative care practices, their self-reported experience with relevant clinical skills, and their attitudes toward palliative medicine as a field. Likert scale questions were divided into two categories: student perception of level of preparation and overall expectations of physicians regarding palliative care practices.

To ensure content validity and relevance to the target population, many survey items were adapted from validated questionnaires used in published studies on palliative care. Notable sources included Nakazawa et al., Yamamoto et al., Miranda et al., and Lopez-Garcia et al. [16-19]. Questions from these studies were selectively adapted to better fit the context of medical student education and to reflect the competencies expected at different levels of training. For instance, questions assessing knowledge of palliative care philosophy, patient communication, and clinical management strategies were modified to reflect students' learning stages and anticipated practical experience in palliative care. Please see Appendices for the question breakdown.

Inclusion criteria were undergraduate medical students of all levels of training at the University of Central Florida College of Medicine. Exclusion criteria were any graduate-level medical students (i.e., residents and fellows), attendings or other health professionals (i.e., nurses, nursing assistants, and physician assistants), and undergraduate medical students from other institutions.

For data analysis, all data other than the year of enrollment in medical school were de-identified. Proportions of correct responses for true/false questions and response choices for the Likert scale questions were calculated. The groups were analyzed using independent t-tests to compare the proportion of correct responses in the various domains.

## Results

Four hundred eighty surveys were electronically distributed, and 125 responses were collected, resulting in a response rate of 26% (N = 125). Of these responders, 39 respondents did not provide their year of training and, thus, received limited analysis, resulting in n = 86 (69%). This group of students was examined for outlying characteristics, which did not show major differences.

Of the responses generated (n = 86), M1s accounted for n = 24 (28%), M2s accounted for n = 28 (33%), M3s accounted for n = 14 (16%), and M4s accounted for n = 20 (23%). To compare scores for true/false questions, respondents were grouped into preclinical students (M1s and M2s), n = 52 (60%), and clinical students (M3s and M4s), n = 34 (40%). The percentage of correct scores in each domain of questions among the preclinical and clinical students is listed in Table 1.

**Table 1:** Scores for preclinical and clinical students for each survey category.

Domain	Preclinical students (% correct) n = 52 (60%)	Clinical students (% correct) n = 34 (40%)
Philosophy of Palliative Care	82	90
Management of Pain	62	68
Respiratory	40	57
Healthcare Disparities	96	97
Neuropsychiatric Disorders	82	88
Gastrointestinal Symptoms	46	63
Communication and Cultural Influence	98	100

An independent sample t-test resulted in p < 0.001, indicating a statistically significant difference in the performance of preclinical students and clinical students for all domains. Likert scale questions on the level of preparation and overall expectations of physicians regarding palliative care practices are summarized in Tables 2, 3.

**Table 2:** Likert scale questions assessing medical students' self-perceived level of preparation.

N = 86	I feel prepared to discuss withholding or withdrawing life-sustaining treatment in the ICU (n (%))	Dealing with end-of-life topics with patients is anxiety-provoking for me (n (%))	I would benefit from more palliative care training (n (%))
Strongly agree	2 (2%)	14 (16%)	71 (83%)
Somewhat agree	5 (6%)	31 (36%)	12 (14%)
Neither disagree nor agree	12 (14%)	16 (19%)	3 (3%)
Somewhat disagree	29 (34%)	19 (22%)	0 (0%)
Strongly disagree	38 (44%)	6 (7%)	0 (0%)

**Table 3:** Likert scale questions assessing medical students' expectations of physicians regarding palliative care practice.

N = 86 (26%)	Doctors have a responsibility to help patients at the end of life prepare for death (n (%))	Doctors have a responsibility to provide bereavement care to the patient's family members after death (n (%))	In order to provide the best end-of-life care for a patient, a physician should be emotionally uninvolved with his or her patient (n (%))
Strongly agree	70 (81%)	39 (45%)	2 (2%)
Somewhat agree	12 (14%)	29 (34%)	14 (16%)
Neither disagree nor agree	4 (5%)	11 (13%)	17 (20%)
Somewhat disagree	0 (0%)	7 (8%)	28 (33%)
Strongly disagree	0 (0%)	0 (0%)	25 (29%)

## Discussion

This study sought to assess medical students' knowledge regarding PPC knowledge in six domains (Philosophy of Palliative Care, Management of Pain, Respiratory, Neuropsychiatric Disorders, Gastrointestinal Symptoms, and Communication and Cultural Influence) as well as attitudes toward performing basic palliative care and the role of the physician in end-of-life care. Overall, respondents in all four years performed very well in the communication and healthcare disparities categories. This may indicate that there is adequate coverage of these topics in the preclinical years that is retained or reinforced throughout the clinical years. Respondents also performed well in the philosophy and psychiatric categories (>80% correct). It should be noted that there was a greater difference in the mean percent correct between preclinical and clinical students in these categories (8% and 7%, respectively). Given that the difference in scores between preclinical and clinical students was shown to be statistically significant (p < 0.001), it is possible that there is coverage of these topics in the clinical years that is further developed during the clinical years. This may speak to the utility of experiential learning in the competencies being assessed in these categories (i.e., understanding the goals of palliative care and managing delirium).

Lastly, respondents showed the poorest performance (40%-60% correct) in the gastrointestinal and pain categories, again with a notable difference between the average percent correct between preclinical and clinical students (16% and 11%, respectively). This could represent inadequate coverage of the management of pain and gastrointestinal conditions in the palliative care setting in preclinical education, which is only partially developed during clinical education. Lower scores after clinical education in these categories compared to other categories suggest that there may not be adequate coverage of these topics during the clinical years. It should be noted that students who did not indicate their year of training tended to have higher percentages of correct answers in the psychology domain.

Students believe these roles are important and part of the physician's responsibilities. Regarding students' perceptions of the role of a physician regarding palliative care, the vast majority agreed that part of a physician's role is to prepare the patient for death (95%) and provide bereavement care to the patient's family (79%). Despite believing that physicians have a responsibility to perform basic palliative care, a majority of students indicated that they did not feel prepared to discuss withholding or withdrawing end-of-life care (77%) and that discussing end-of-life topics was anxiety-provoking for them (52%). While there may be several explanations for students' lack of self-confidence with regard to practicing palliative care, it is likely that it can be in part attributed to the lack of adequate training. This hypothesis is supported by 97% of respondents agreeing that they would benefit from more palliative care training.

The results of this study are significant as medical student knowledge of PPC is infrequently addressed in the literature. Potential solutions to this issue could include providing greater coverage of palliative care in preclinical curricula, requiring palliative care experiences during core clinical rotations, offering clinical palliative care electives, or utilizing short asynchronous courses for medical students. While these are proposed solutions based on review of the literature, it should be acknowledged that the gaps in knowledge of PPC among medical students may not be solely attributed to a lack of curricular coverage, and investigation of additional causes is outside the scope of this study.

Strengths of this study include the use of validated surveys and its cost-effectiveness. Limitations of this study include social desirability response bias, lack of incentivization, suboptimal response rate, data from only one institution, and potential for survey fatigue due to the length of the survey. Students who did not indicate their year of training tended to have higher percentages of correct answers in the psychology domain, which may have also impacted our conclusions.

## Conclusions

The findings of this study demonstrate that there is a gap between medical students' perception of the importance of palliative care and their level of knowledge and confidence in providing PPC. This is important given that the vast majority of physicians across many specialties will encounter a patient in need of palliative care services in their practices. Knowing how to perform basic PPC and when to refer a patient to a subspecialist can decrease disease burden and aid in maintaining quality of life.

The results of this study are significant as medical student knowledge of PPC is infrequently addressed in the literature. Future studies could be strengthened by incentivization, consolidating the survey while still being able to adequately assess each domain, and requiring participants to identify their year of schooling. Further, data collected from this and future studies can then be used to inform the development of interventions to increase integration of PPC into medical school curricula.

## Appendices

**Table 4:** Survey questions with the type, associated domain, and source. To ensure content validity and relevance to the target population, many survey items were adapted from validated questionnaires used in published studies on palliative care. Notable sources included Nakazawa et al., Yamamoto et al., Miranda et al., and Lopez-Garcia et al. [16-19]. Relevant permissions were obtained from the original publishers.

Question	Type of question	Domain	Source of question
1. Palliative care should only be provided for patients who have no curative treatments available.	True or false	Philosophy of Palliative Care	Nakazawa et al. (2009) [16]
2. Palliative care should not be provided at the same time as cancer treatments.	True or false	Philosophy of Palliative Care	Lopez-Garcia et al. (2022) [19]
3. Palliative care is synonymous with terminal care.	True or false	Philosophy of Palliative Care	Yamamoto et al. (2013) [17]
4. One of the goals of pain management is to get a good night's sleep.	True or false	Management of Pain	Nakazawa et al. (2009) [16]
5. When opioids are taken on a regular basis, non-steroidal anti-inflammatory drugs should not be used.	True or false	Management of Pain	Nakazawa et al. (2009) [16]
6. It is necessary to use a laxative together with oral opioids because most patients who take opioids experience constipation.	True or false	Management of Pain	Yamamoto et al. (2013) [17]
7. Long-term use of opioids can often induce addiction.	True or false	Management of Pain	Nakazawa et al. (2009) [16]
8. Use of opioids does not influence survival time.	True or false	Management of Pain	Nakazawa et al. (2009) [16]
9. Morphine is used safely in a patient with renal failure.	True or false	Management of Pain	Yamamoto et al. (2013) [17]
10. Opioid rotation or switching should be considered when it is difficult to increase the dose of a given drug due to adverse effects.	True or false	Management of Pain	Yamamoto et al. (2013) [17]
11. Morphine is effective for dyspnea.	True or false	Respiratory	Yamamoto et al. (2013) [17]
12. When opioids are taken on a regular basis, respiratory depression will be common.	True or false	Respiratory	Nakazawa et al. (2009) [16]
13. Oxygen saturation levels can predict the level of dyspnea in an end-stage pulmonary or cardiac patient.	True or false	Respiratory	Nakazawa et al. (2009) [16]
14. Anticholinergic drugs are effective for alleviating bronchial secretions of dying patients.	True or false	Respiratory	Nakazawa et al. (2009) [16]
15. Multi-factorial causes of delirium such as medications and physical illness should be explored.	True or false	Neuropsychiatric Disorders	Yamamoto et al. (2013) [17]
16. Some dying patients will require continuous sedation to alleviate suffering.	True or false	Neuropsychiatric Disorders	Nakazawa et al. (2009) [16]
17. Morphine is often a cause of delirium in terminally ill cancer patients.	True or false	Neuropsychiatric Disorders	Nakazawa et al. (2009) [16]
18. When a patient has a high level of psychological distress, clinicians should examine whether the patient has suicidal ideation.	True or false	Neuropsychiatric Disorders	Yamamoto et al. (2013) [17]
19. An anxiolytic is one of the useful medications for patients with psychological distress.	True or false	Neuropsychiatric Disorders	Yamamoto et al. (2013) [17]
20. At the terminal stages of cancer, higher caloric intake is needed compared to the early stages.	True or false	Gastrointestinal Symptoms	Nakazawa et al. (2009) [16]
21. Central venous lines are needed for comfort medications for patients without peripheral intravenous access.	True or false	Gastrointestinal Symptoms	Nakazawa et al. (2009) [16]
22. Steroids can improve appetite among patients with advanced cancer.	True or false	Gastrointestinal Symptoms	Lopez-Garcia et al. (2022) [19]
23. Intravenous infusion is not effective for alleviating dry mouth in dying patients.	True or false	Gastrointestinal Symptoms	Lopez-Garcia et al. (2022) [19]
24. When physicians convey bad news, they should ask about the patient's concern and understanding of the disease.	True or false	Communication and Cultural Influence	Yamamoto et al. (2013) [17]
25. Culture can influence a patient's healthcare decision-making.	True or false	Communication and Cultural Influence	N/A
26. A professional interpreter is not necessary if family members are available and able to translate.	True or false	Communication and Cultural Influence	N/A
27. Palliative care services are often underutilized by members of racial and ethnic minority groups.	True or false	Healthcare Disparities	N/A
28. Equitable access to healthcare is associated with English proficiency.	True or false	Healthcare Disparities	N/A
29. What is your current year of training? If taking a gap year, please select the year of training completed.	Multiple choice	N/A	N/A
30. Have you completed an elective rotation in palliative care?	Yes or no	N/A	N/A
31. Have you completed an elective rotation in any subspecialty of oncology?	Yes or no	N/A	N/A
32. Do you have experience working or volunteering in a hospice or palliative care unit BEFORE medical school?	Yes or no	N/A	N/A
33. Do you have experience working or volunteering in a hospice or palliative care unit DURING medical school?	Yes or no	N/A	N/A
34. How often have you experienced a collaboration with palliative care in clinical practice?	Likert scale	N/A	Miranda et al. (2019) [18]
35. Have you personally participated in a discussion about withdrawing life-sustaining treatment with a patient with serious illness?	Yes or no	N/A	N/A
36. Have you personally participated in a multidisciplinary team meeting (e.g., nursing, social work, and specialists) for a patient with serious illness?	Yes or no	N/A	N/A
37. Have you participated in advanced care or goals-of-care planning with your personal friends or family? - Selected choice	Yes or no	N/A	N/A
If yes, optional text box if willing to provide additional details:	Free response	N/A	N/A
38. How to lead a family meeting	Yes or no	N/A	Miranda et al. (2019) [18]
39. How to utilize ethical frameworks in end-of-life care decision-making	Yes or no	N/A	Miranda et al. (2019) [18]
40. How to engage in shared decision-making with patients regarding their values and preferences for end of life	Yes or no	N/A	Miranda et al. (2019) [18]
41. How to communicate to a surrogate or family member that a patient is dying	Yes or no	N/A	Miranda et al. (2019) [18]
42. I feel prepared to discuss withholding or withdrawing life-sustaining treatment in the ICU	Likert scale	N/A	Miranda et al. (2019) [18]
43. I have participated in operations/procedures and worried whether they were aligned with patient values or goals	Likert scale	N/A	Miranda et al. (2019) [18]
44. Doctors have a responsibility to help patients at the end of life prepare for death	Likert scale	N/A	N/A
45. Doctors have a responsibility to provide bereavement care to the patient's family members after death	Likert scale	N/A	N/A
46. Palliative care can be administered concurrently with life-prolonging treatments	Likert scale	N/A	Miranda et al. (2019) [18]
47. In order to provide the best end-of-life care for a patient, a physician should be emotionally uninvolved with his or her patient	Likert scale	N/A	Miranda et al. (2019) [18]
48. Dealing with end-of-life topics with patients is anxiety-provoking for me	Likert scale	N/A	N/A
49. I would benefit from more palliative care training	Likert scale	N/A	N/A
